# Production of Protein Hydrolysates from Cod Backbone Using Selected Enzymes: Evaluation of Antioxidative and Antimicrobial Activities of Hydrolysates

**DOI:** 10.3390/md23030125

**Published:** 2025-03-13

**Authors:** Dimitra Marinou, Charlotte Jacobsen, Davide Odelli, Krystalia Sarigiannidou, Ann-Dorit Moltke Sørensen

**Affiliations:** 1 Research Group for Bioactives–Analysis and Application, National Food Institute (DTU Food), Technical University of Denmark, DK-2800 Kgs. Lyngby, Denmark; dimi24_96@yahoo.gr (D.M.); chja@food.dtu.dk (C.J.); 2 Departamento de Tecnologia de Alimentos, Universidade Federal de Viçosa (UFV), Viçosa 36570-900, MG, Brazil; 3 Research Group for Food Production Engineering, National Food Institute (DTU Food), Technical University of Denmark, DK-2800 Kgs. Lyngby, Denmark; krystaliasari@gmail.com

**Keywords:** marine rest raw material, bioactive peptides, cod side-stream, utilization, valorization

## Abstract

In the fish industry, up to 70% of all fish end up as side-streams such as backbones, heads, and viscera. To reduce the quantities of side-streams, a higher utilization degree of fish is needed. The aim of this study was to use cod backbone for an enzymatic production of bioactive hydrolysates with antioxidative and/or antimicrobial properties. Three different enzymes were applied (Alcalase, Neutrase, and Protamex), and hydrolyses were carried out within the enzyme’s optima for pH and temperature for 0.5–6 h. The efficiency of the enzyme treatment was evaluated based on the protein extraction yield (PEY), the degree of hydrolysis (DH), and antioxidant activity using two different in vitro assays (1,1-diphenyl-2-picrylhydrazyl (DPPH) radical scavenging and iron chelation) and antimicrobial activity determined by minimum inhibitory concentration (MIC) and disk diffusion assays. Selected hydrolysates showing activity were evaluated with respect to amino acid composition and molecular weight. Alcalase-treated samples had the highest PEY (3 h, 63.5 ± 4.5%) followed by Protamex-treated samples (3 and 6 h; 51.9 ± 5.5% and 56.5 ± 4.5%); the lowest PEY was obtained with Neutrase (3 and 6 h; 30.4 ± 1.9% and 34.7 ± 3.4%). No clear relationship was observed between the PEY and DH. All hydrolysates had antioxidant activities. For radical scavenging activity, Protamex-treated hydrolysate showed the lowest IC_50_ (6 h, 2.1 ± 0.1 mg powder/mL) and had a molecular weight <10 kDa, whereas for iron chelation activity, the control samples (no enzyme added but heat-treated) showed a similar or lower IC_50_ with molecular weights of 200–10 kDa. Amino acid composition measured on selected hydrolysates suggested that not only the composition of amino acid but also sequence and size influence the properties. None of the hydrolysates showed antimicrobial activity. In summary, the results showed that protein hydrolysates with antioxidant activity can be produced from the cod backbone, which makes it possible to utilize this side-stream generated in the fish industry.

## 1. Introduction

In 2022, the global fisheries (wild and aquaculture) reached 185 million tonnes, where approx. 165 million tonnes (89%) were used for human consumption. An annual increase of 3% in the number of fish used for human consumption has been observed from 1961 to 2022 [1]. This high increase in marine production is caused by increased demand by the continuously growing global population. This will result in an increasing number of marine side-streams generated from the fish industry [2]. The generated side-streams that derive from the fish industry can account for up to 70% of all fish and include the head, skin, trimmings, fins, backbones, viscera, and fillet cut-offs [2,3,4,5]. These side-streams are often used as agricultural fertilizers and animal feed or even wasted; however, this may not always be feasible or profitable [5] and also leaves their broad bio-functionality spectrum unexploited [3,4]. In Denmark, a large proportion of the fish side-streams was fed to minks, but, due to COVID-19, mink farming has been abandoned in Denmark. Hence, further utilization and new application opportunities for these side-streams are necessary.

The side-streams deriving from marine sources have high nutritional and biological value due to the presence of proteins (bioactive peptides, collagen, gelatin, enzymes, etc.), fish oils, minerals, and more [3,5,6,7,8,9]. Due to their valuable content, great attention has been drawn to the application possibilities of marine side-streams during the last two decades. Low-cost opportunities can arise from exploiting those attributes for developing food ingredients, nutraceuticals, pharmaceuticals, and cosmetics with functional properties. At the same time, a large and rational utilization of marine side-streams could contribute both to the environment and to more sustainable production processes instead of ending up as waste, which is the case for some fish product production sites [5,10]. Kaushik et al. [11] highlighted that efficient biotechnological methods for valorizing marine side-streams to fish protein hydrolysates (FPHs) could enable future applications of the fish protein hydrolysates as a sustainable source of protein.

Earlier studies have reported that antimicrobial and antioxidant peptides can be produced from fish raw materials. Antimicrobial peptides are usually medium-sized peptides, 12–50 amino acid residues, while the antioxidant peptides are usually slightly smaller size peptides, 3–20 amino acid residues [2,12,13]. Peptides with antimicrobial and/or antioxidant activities have been produced from various fish and fish side-streams with different enzymes and hydrolysis times in the range from 10 min to 20 h [2,14,15,16,17,18,19,20,21,22]. Although several studies have evaluated bioactive compounds produced by enzyme hydrolysis from various side-streams from, e.g., swim bladders of miiuy croaker [23], bluefin leatherjacket skin [24], and skipjack tuna scales [25], none of these studies carried out hydrolysis without enzyme addition to evaluate the effect of the enzyme addition nor did they evaluate both the antioxidative and antimicrobial activity of the FPH produced.

Enzymatic hydrolysis on different fish and fish side-streams has been performed and reported with bioactive and/or functional properties, but only a few studies were performed on the cod backbone as substrate for the enzymatic hydrolysis. From a mix of cod backbone and viscera, a 23–57% higher amount of FPH was obtained using Neutrase (exo-peptidase) compared to Flavorzymes (endo-peptidase) [26]. Earlier, antioxidative FPH has been produced by the enzymatic hydrolysis of cod backbone using Protamex (0.1% based on raw material weight, 55 °C, 10–60 min). Higher antioxidant activity measured by DPPH radical scavenging activity was obtained by the enzymatic hydrolysis of fresh cod backbone compared to backbone, which was stored frozen prior to the hydrolysis. Moreover, a tendency to increased radical scavenging activity (DPPH) with increased hydrolysis time, 10 min to 60 min, was observed [20]. In addition, Jafarpour et al. [27] produced antioxidative FPH from cod backbone using different proteases (Alcalase and Neutrase) added individually or sequentially. In these studies, the full impact of adding the enzyme was not fully evaluated since a control without enzyme addition but exposed to the same conditions as the enzyme-treated samples was not included. This study aimed to produce FPH (mixture of peptides) from cod backbone, a solid side-stream generated in the cod filleting process, with antimicrobial and/or antioxidant properties, intended for high-quality animal feed, food, or other applications. Different enzymes (Alcalase, Neutrase, and Protamex) with different hydrolysis conditions were used to produce hydrolysates. The hydrolysis time was varied between 0.5 and 6 h for the different enzymes, and the pH and temperature were set within the optima for the individual enzymes applied. The selection of enzymes and hydrolysis conditions was based on a literature review, and they were in similar ranges as those of other hydrolysis studies carried out on different marine side-streams (bones/backbones) using these enzymes [11,27,28].

In addition, controls were included to evaluate the effect of the enzyme addition. Controls were treated as the enzyme-treated samples, i.e., similar pH, temperature, and time without the addition of enzyme. For one of the Alcalase treatments, the effect of preheating before enzyme treatment, i.e., the inactivation of endogenous enzymes, was also investigated. The experimental design for the hydrolysis with the different enzymes is summarized in Table 1.

## 2. Results

The efficiency of the hydrolysis process was evaluated by the protein extraction yield (PEY) and the degree of hydrolysis. The enzymes cleave the protein and increase the solubility of the protein, which will increase the protein extracted in the hydrolysates.

### 2.1. Protein Extraction Yield (PEY)

**Figure 1:**
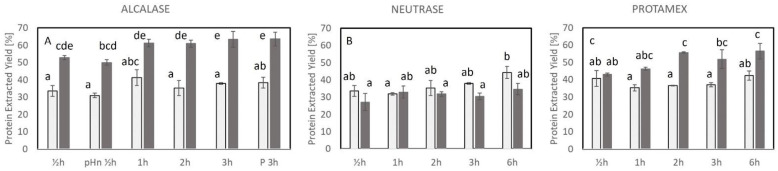
Protein extraction yield, PEY [%], obtained in lyophilized hydrolysates after hydrolysis with different enzymes. (**A**) Alcalase-treated samples, (**B**) Neutrase-treated samples, and (**C**) Protamex-treated samples. Light bars: control, no enzyme added; dark grey bars: enzyme-treated samples. The bars indicate the average of duplicate measure and STD. Different letters (a–e) indicate significant differences between samples. For conditions of the treatments, refer to Table 1. pHn ½h: no pH adjustment prior to hydrolysis (Alcalase ½h); P 3h: preheating prior to hydrolysis (Alcalase 3h).

Out of the three enzymes, the highest amount of protein from the cod backbones was recovered using Alcalase (1–3 h, >60%) for the enzyme hydrolysis (Figure 1). This is explained by the higher amount of supernatant and protein content for those samples (Table S1).

The PEY of the controls (no enzymes added) for Alcalase-treated samples was lower (31–41%) and significantly different from the PEY obtained of the corresponding Alcalase-treated samples irrespective of the hydrolysis time (50–64%, Figure 1A). The PEYs obtained with the controls were at similar levels with no significant differences between the different heating times (½–3 h). The PEY increased from ½ h hydrolysis (50–53%) to 1 h of hydrolysis (61 ± 2.1%); however, the PEYs obtained after 1, 2, and 3 h of hydrolysis were not significantly different (61–63%, *p* > 0.05). The PEYs for samples hydrolyzed for ½ h with Alcalase with (A ½ hE) and without pH (A pHn ½ hE) adjustment were not significantly different. Moreover, it was observed that 3 h and P 3h, i.e., the Alcalase-treated sample without and with prior preheating, did not differ significantly, which indicates that the preheating did not influence the enzymatic activity for Alcalase-treated samples with a hydrolysis time of 3 h and that the presence or influence of endogenous enzyme activity on the PEY was limited.

The PEY obtained with Neutrase was in the range of 27–44% (Figure 1B). The PEYs obtained for the controls were in a similar range (32–44%) as the Neutrase-treated samples, and no significant differences were observed. Thus, the PEY was not affected by enzyme addition and hydrolysis time ½–6 h (60 °C, pH 8).

The PEY obtained after hydrolysis with Protamex was between 43 and 57% (Figure 1C). A higher PEY was obtained for Protamex-treated samples hydrolyzed for a longer time (2 h to 6 h) than for a shorter time (0.5–1 h). The addition of Protamex only increased the PEY compared to the corresponding controls when the hydrolysis times were longer than 1 h. The PEY obtained for the controls (50 °C, pH 6.5) was similar (35–42%) and independent of the heating time (½–6 h).

### 2.2. Degree of Hydrolysis (DH)

**Figure 2:**
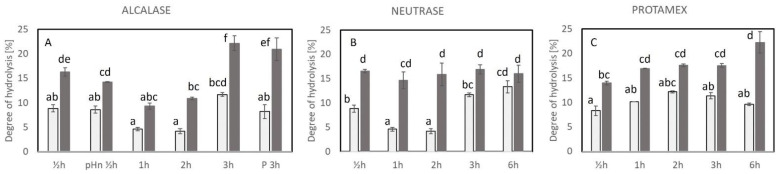
Degree of hydrolysis [%] obtained after hydrolysis with different enzymes. (**A**) Alcalase-treated samples, (**B**) Neutrase-treated samples, and (**C**) Protamex-treated samples. Light bars: control, no enzyme added; dark grey bars: enzyme-treated samples. The bars indicate the average of duplicate measure and STD. Different letters (a–f) indicate significant differences between samples. For conditions for the treatments, refer to Table 1. pHn ½h: no pH adjustment prior to hydrolysis (Alcalase ½h); P 3h: preheating prior to hydrolysis (Alcalase 3h).

For an efficient enzyme hydrolysis process, it is expected that DH increases with increased hydrolysis time. In Figure 2, the DH obtained using different enzymes and hydrolysis conditions is shown.

In the Alcalase-treated samples, a significant difference was observed between the control and Alcalase-treated samples for each hydrolysis time except from the 1 h samples (Figure 2A). For Alcalase-treated cod backbone, 3 h of hydrolysis resulted in significantly higher DH than 0.5–2 h of hydrolysis, whereas 0.5 h of hydrolysis with Alcalase resulted in significantly higher DH than 1 and 2 h (Figure 2A). Moreover, DH was almost doubled when the hydrolysis time increased from 1 h to 3 h. The DH obtained after 1 and 2 h of hydrolysis with Alcalase was not significantly different.

The DH obtained after 0.5 h hydrolysis with and without pH adjustment was not significantly different irrespective of whether Alcalase was added or not (½hE vs. pHn_½hE, ½hC vs. pHn_½hC), indicating that the pH adjustment of the cod backbone prior to hydrolysis did not play a major role. Additionally, preheating to inactivate endogenous enzymes prior to the hydrolysis did not result in different DHs as also observed for the PEY with similar amounts.

Concerning the results of the hydrolysis with Neutrase, no correlation between the hydrolysis time and DH was observed (Figure 2B). The DH ranged from 14.7 to 16.9%, and no significant differences were observed for the Neutrase-treated cod backbone. The control samples from 0.5 h had a significantly higher DH than control samples after 1 and 2 h treatment, and control at 6 h had the highest DH compared to controls and significantly different from 0.5 h–2 h heating. Regardless of the samples deriving from the same cod backbone batch, a variation in the amount of the endogenous enzymes in each sample is possible, which could result in different DHs. However, the differences observed with DH for the controls did not affect the PEY (Figure 1B). The comparison of the DH obtained with Neutrase and Alcalase with the same hydrolysis conditions showed that Neutrase, irrespective of the hydrolysis time, only achieved the same DH as Alcalase after 0.5 h of hydrolysis.

The DH of the samples treated with Protamex increased slightly from 0.5 h to 6 h of hydrolysis with the highest DH (22.3 ± 2.2%) after 6 h, whereas 0.5 h of hydrolysis had the lowest DH (14.0 ± 0.4%), Figure 2C. The DH obtained after 1 h, 2 h, and 3 h of hydrolysis remained constant without significant differences. No significant differences were observed for the control samples of Protamex. The Protamex controls had a higher DH for 1 h and 2 h heating than the Alcalase/Neutrase controls (possibly due to the different conditions of treatment, lower temperature, and pH for Protamex controls), which seemed to have influenced the endogenous enzyme activity.

### 2.3. Antioxidative Properties of the Hydrolysates

**Figure 3:**
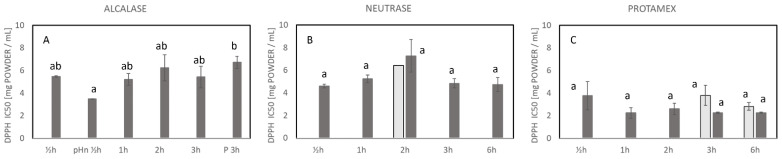
Radical scavenging activity measured by the DPPH assay after hydrolysis with different enzymes and expressed as concentration [mg powder/mL] to reach IC_50._ Light bars: control, no enzyme added; dark grey bars: enzyme-treated samples. No visible bars means that IC_50_ was not reached. (**A**) Alcalase-treated samples, (**B**) Neutrase-treated samples, and (**C**) Protamex-treated samples. The bars indicate the average of duplicate measure and STD. Different letters (a,b) indicate significant differences between samples. For conditions for the treatments refer to Table 1. pHn ½h: no pH adjustment prior to hydrolysis (Alcalase ½h); P 3h: preheating prior to hydrolysis (Alcalase 3h).

**Figure 4:**
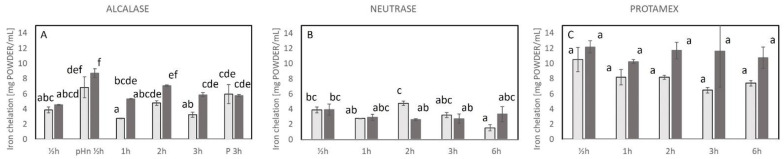
Iron chelation activity measured by the Ferrozine assay after hydrolysis with different enzymes and expressed as concentration [mg powder/mL] to reach IC_50_ (**A**) Alcalase-treated samples, (**B**) Neutrase-treated samples, and (**C**) Protamex-treated samples. The bars indicate the average of duplicate measure and STD. Different letters (a–f) indicate significant differences between samples. For conditions for the treatments, refer to Table 1. Light bars: control, no enzyme added; dark grey bars: enzyme-treated samples. pHn ½h: no pH adjustment prior to hydrolysis (Alcalase ½h); P 3h: preheating prior to hydrolysis (Alcalase 3h).

In the current study, the antioxidative properties of the produced hydrolysates were evaluated by the radical scavenging activity (RSA) and iron chelating activity. Hydrolysates with lower IC_50_ had higher activity due to the lower amount of hydrolysates needed to reach 50% inhibition or activity. All samples hydrolyzed with Alcalase, Neutrase, and Protamex, including all the control samples, exhibited antioxidant activity in the form of RSA (Figure 3) and /or iron chelation activity (Figure 4) at the various times of hydrolysis. Even though a higher amount of sample was used to achieve the IC_50_ for some of the enzyme-treated samples, all of them had antioxidant properties.

In general, the enzyme treatment improved RSA, where IC_50_ was affected by the different enzymes applied. At three of the treatments, the control reached IC_50_ (N_2hC, P_3hC, and P_6hC), whereas for the rest of the control treatments, IC_50_ was not reached (Figure 3). The hydrolysates produced with Protamex (2.2–3.8 mg/mL) had higher RSA than Alcalase (3.5–6.7 mg/mL) and Neutrase (4.6–7.2 mg/mL)-produced hydrolysates. Unexpectedly, all the different hydrolysis times (0.5–6 h) with either Alcalase (Figure 3A), Neutrase (Figure 3B), or Protamex (Figure 3C) did not affect the RSA significantly. However, a significantly better RSA was observed for A_pHn ½hE (no pH adjustment) compared with A_P 3hE (preheating prior hydrolysis), i.e., the inactivation of endogenous enzymes before hydrolysis (Figure 3A). The RSA obtained for hydrolysates produced with the different enzyme treatments showed no correlation with DH.

The iron chelating activity (IC_50_) with the different treatments was in the range of 2.8–8.7 mg/mL for Alcalase, 1.5–4.8 mg/mL for Neutrase, and 6.5–12.2 mg/mL for Protamex and their controls. In contrast to RSA, the iron chelating activity of the hydrolysates was not improved with the enzyme treatment (Figure 4). The controls performed similarly to the enzyme-treated samples, and, for a few samples, the controls were even significantly better than the Alcalase-produced hydrolysates, A_pHn ½h, A_1h and A_3h (Figure 4A). The treatment resulting in the highest iron chelating activity was the control for the Neutrase treatment after 6 h (1.5 ± 0.1 mg/mL).

#### 2.3.1. Total and Free Amino Acids

The content of total and free amino acids was measured in three selected hydrolysates: the Protamex-treated sample after 6 h (P_6hE), the control for the Protamex treatment (P_6hC), and the control for the Neutrase treatment after 6 h (N_6hC), Table 2. The hydrolysates were selected based on their antioxidant activity, where the two from the Protamex had similar activities for both RSA and iron chelation with (P_6hE) and without (P_6hC) the enzyme Protamex, and they also had the highest RSA and among the lowest iron chelation activity. N_6hC had no RSA and the highest iron chelation activity.

For the three selected samples, the sum of total amino acids was not significantly different, whereas the sum of the free amino acids was significantly different. The highest content of the free amino acids, both individual amino acids and the sum, was obtained in the Protamex-hydrolyzed sample (P_6hE) followed by the controls for Protamex (P_6hC, pH 6.5, 50 °C) and Neutrase (N_6hC, pH 8, 60 °C). For the two controls, the differences in the free amino acids did not show the same differences in DH, where a higher DH was obtained with N_6hC than P_6hC (Figure 2). This may be explained by different cleavages of endogenous enzymes at different temperatures and pH that resulted in different molecular sizes of peptides and free amino acids in the samples. This has to be further evaluated.

#### 2.3.2. Molecular Size (Selected Hydrolysates)

The SEC-MALS chromatography of the controls and the samples after each enzyme treatment displayed 6–7 elution peaks, and the molecular weight distribution was divided in five bands depending on peptide fractions estimated molecular weight (Mw), which corresponded to Mw (kDa) > 200, 200–100, 100–10, 10–1, ≤1. The relative proportions (%) of each band for the hydrolysates obtained with and without enzyme addition are shown in Table 3.

As can be seen from the table, the controls (no enzymes added prior to the heat treatment) presented all of the peptide fraction components in the bands 200–100 kDa and 100–10 kDa and a 24.4% in the molecular weight band 10–1 for one of the controls (N_6hC). On the other hand, in the samples with Neutrase (6h, N_6hE) and Alcalase (3 h) with or without prior preheating (A_P_3hE and A_3hE), peptide fractions with estimated Mw > 200 kDa were also found. These high molecular weight fractions were considered as partially hydrolyzed peptides, which, however, were only present in low relative proportions, being 1.8, 0.3, and 0.3% for the N_6hE, A_P_3hE, and A_3hE, respectively (Table 3). Moreover, N_6hE presented more than 80% of its peptides in the bands 100–10 and 10–1 kDa, while A_P_3hE and A_3hE presented more than 95% of their fraction with peptides of Mw ≤ 1 kDa, thus representing the enzymatic treatments with the highest amount of low-molecular-weight peptides. Notably, sample P_6hE (treated with Protamex for 6h) was only composed of peptides with Mw < 10 kDa, with fractions ≤ 1 kDa being the main components with a relative proportion of 85%. These results suggest that the extraction and processing conditions of fish protein produce a wide mixture of peptides and that the hydrolysis by Alcalase, Neutrase, and Protamex mainly results in low-molecular-weight peptides (<10 kDa). Here, Alcalase (A_P_3hE and A_3hE; 95 and 97%) followed by Protamex (P_6hE; 84.6%) had the highest fraction of peptides with Mw ≤ 1kDa compared with Neutrase (N_6hE; 15.9%). This is also in line with the higher PEY and DH for these samples.

### 2.4. Antimicrobial Activity

The first hydrolysates were produced with Alcalase, and their antimicrobial activity was examined using the MIC assay. However, no microbial growth occurred for any of the Alcalase samples. Hence, the Alcalase-treated samples and controls exhibited no antimicrobial activity in the concentration range evaluated (10 and 100 mg/mL) under the conditions evaluated. In addition, the disk diffusion assay was conducted for the Alcalase samples, which is a different method to determine the antimicrobial activity; however, no inhibition zones were created for the evaluated concentration (10 and 100 mg/mL). After these negative results, all hydrolysates obtained after Alcalase, Neutrase, and Protamex treatment were evaluated with the disk diffusion assay at higher concentrations (200 mg/mL). None of the produced hydrolysates formed inhibition zones; hence, no antimicrobial activity was detected for the produced hydrolysates.

## 3. Discussion

### 3.1. The Produced Fish Protein Hydrolysates: PEY and DH

In the current study, a PEY of 50–64% was obtained using Alcalase at different hydrolysis times. Another study with Alcalase-hydrolyzed cod backbone (pH 7.4, 50 °C, 1.5% E/S, 3 h) resulted in a nitrogen recovery of 69% [28], which is slightly higher than what was obtained in the current study (63%, 3 h) and might be explained by the higher Alcalase concentration used in the study by Jafarpour et al. [28]. However, the lower pH and temperature applied in that study can also have had an impact on the PEY. Moreover, the cod in the previous study was also much larger and may have had more meat on the bones than those from Greenland in the current study, which may explain the differences observed with Alcalase-hydrolyzed cod backbone. However, Alcalase treatment (3%, pH 7.5, 50 °C, 3 h) head and backbone blend obtained from catfish (*Pangasius hypophthalmus*) resulted in similar protein recovery (60.4% ± 5.7) as the current study despite different side-stream materials and hydrolysis conditions, enzyme concentration, temperature, and pH [29].

For Neutrase-treated cod backbone, a larger deviation was obtained from previously published results, since Jafarpour et al. [28] obtained a nitrogen recovery of 50% using Neutrase (pH 7.4, 50 °C, 1.5% E/S, 3 h), which was 1.7-fold higher than the current study (PEY 30%) after 3 h of hydrolysis. Again, the different enzyme concentrations, lower pH, and temperature applied and/or amount of meat left on the bones can have had a greater impact for the efficiency of Neutrase compared to Alcalase. In the current study, there was no preheating to inactivate endogenous enzymes prior to the hydrolysis. Knowing that fish tissue contains high quantities of proteases inhibitors [30], this could explain the similar PEY of controls and Neutrase-treated samples, where the cleavage of the peptide bonds in the current study was facilitated via heat instead of the enzymatic activity. When comparing results from Alcalase treatments with those from Neutrase treatments, it seems that endogenous protease inhibitors were not able to inhibit Alcalase due to the significantly higher PEY observed for Alcalase-treated samples and controls compared to Neutrase-treated samples and controls. Looking at the DH obtained using Alcalase, the results contradict earlier findings reported by Sila et al. [22] obtained with Alcalase-treated barbel muscles, where a steady increase in the DH over time was observed. A hydrolysis time of 0.5 h and 2 h resulted in a DH of ~10% and ~16%, respectively [22]. The different substrates used could explain these differences. Moreover, the amount of Alcalase used to hydrolyze the barber muscle was difficult to compare as different units were used (1:1 U/mg enzyme/protein ratio). Additionally, an earlier study with Alcalase-treated cod backbone (pH 7.4, 50 °C, 1.5% E/S, 3 h) resulted in a DH of 36 ± 0.03% [28]. This was in correspondence with the finding that also the PEY was higher in that study compared to ours as previously discussed. However, the Alcalase treatment (3%, pH 7.5, 50 °C, 3 h) head and backbone blend obtained from catfish (*Pangasius hypophthalmus*) resulted in a similar DH (22.9 ± 1.6%) as the current study despite different side-stream materials and hydrolysis conditions, enzyme concentration, temperature, and pH [29]. The obtained DH results for Neutrase-treated hydrolysates contradict the findings obtained by Jafarpour et al. [28], where Neutrase-treated cod backbone (pH 7.4, 50 °C, 1.5% E/S, 3 h) resulted in a DH of 27 ± 0.1%, which is 1.6-fold higher. Again, the enzyme concentration was higher, and the substrate was different, which may explain both the higher DH and protein recovery obtained with Neutrase. Moreover, a lower pH and temperature were applied in their study, which also can impact the hydrolysis. Moreover, Benjakul and Morrissey [31] obtained significantly higher proteolytic activity with Alcalase than Neutrase-treated pacific whiting solid side-streams as substrate (Alcalase: pH 9.5, 60 °C; Neutrase: pH 7.0, 55 °C, 0.5 h) at equal enzyme concentrations (20 AU/kg), which also contradict the DH obtained in the current study after 0.5 h treatment. However, in their study, the pH was maintained during hydrolysis, which clearly influenced the hydrolysis and proteolytic activity of the enzymes [31]. In multiple studies, the pH was constantly monitored and adjusted to optimum throughout the hydrolysis of the different marine raw materials [17,22,32]. For the current study, the cod backbones were hydrolyzed without constant monitoring and adjusting pH. The DH results obtained for Protamex-hydrolyzed hydrolysates are in accordance with the results obtained from Šližyte et al. [20], who hydrolyzed frozen and fresh minced/chopped cod backbones using Protamex for 10, 25, 45, and 60 min (55 °C, 0.1% Protamex by the weight of raw material) [20]. The samples were not preheated, and the enzyme inactivation after the hydrolysis was performed by microwave heating >90 °C for 5 min. They observed a minor increase in DH over time, which reached 21.4–24.3% from 10 min to 1 h of hydrolysis, whereas in this study, a DH of over 22% was only achieved at 6 h of hydrolysis. This could be due to the variation of the present endogenous enzymes in the different marine raw materials, the soluble muscle-to-bone ratio in the samples, or to the different hydrolysis conditions, e.g., pH, hydrolysis temperature, and the inactivation of enzyme. In contrast, You et al. [21] have reported similar levels of DH for Protamex-hydrolyzed loach for 2 h and 6 h, 18% and 28%, respectively.

In the current study, it is shown that the PEY and DH can be affected by the different enzymes and hydrolysis conditions, which is supported by earlier reported results using different enzymes (alkaline protease, Protamex, and Flavourzyme) and side-streams from different fish species (trout (*Onchorynchus mykiss*), anchovy (*Engraulis encrasicolus*), whiting (*Merlangius merlangus*)) for the optimization of the hydrolysis conditions for the production of protein hydrolysates [33].

### 3.2. Antioxidant Activities of the Fish Protein Hydrolysates

The antioxidant activity (DPPH RSA) obtained for hydrolysates produced with the different enzyme treatments showed no correlation with the DH, which disagrees with the result obtained by Ktari et al. [34]. The examination of the DH results obtained from different protease treatments including the Alcalase (0.5 U/mg protein, pH 8, 50 °C, 1–9 h) of cuttlefish (*Sepia officinalis*) by-products and antioxidant activity showed a positive correlation between the DH and antioxidant activity [34]. In addition, DPPH RSA has also been observed to slightly increase with increasing DH for Alcalase-treated cod backbone and round scad muscle [20,35], whereas for yellow stripe trevally, a decrease in DPPH RSA was observed with increasing DH [36]. No correlations between DH and DPPH RSA have also been observed for Protamex-hydrolyzed loach meat [21]. The different observations for the DH and DPPH RSA connection are assumed to be ascribed to different experimental parameters such as substrate, enzyme concentrations, etc., which will result in different lengths and molecular structures of peptides in the hydrolysates and, in turn, in different antioxidant activities. High DPPH RSA activity has been reported for marine raw materials at 80–90% inhibition using different proteases, where actual concentrations of the hydrolysates were evaluated instead of IC_50_ [21,37]. The 90% inhibition was obtained with hydrolysates obtained from Protamex-hydrolyzed loach for 4–20 h, where the evaluated hydrolysates had a concentration of 40 mg protein/mL [21]. In the current study, the most efficient hydrolysate for DPPH RSA was obtained with Protamex 1 h–6 h of hydrolyses (IC_50_ 2.2–2.6 mg powder/mL), which is a much higher protein concentration than observed for hydrolyzed loach. However, the loach hydrolysates are obtained only from hydrolyzed meat, whereas ours are a mix of fish bones and meat, which may explain the differences.

The Iron chelating activity (IC_50_) of the produced hydrolysates with the different treatments (2.8–8.7 mg/mL for Alcalase, 1.5–4.8 mg/mL for Neutrase, and 6.5–12.2 mg/mL for Protamex and their controls) showed much lower iron chelating activity than earlier reported for Alcalase and Neutrase-treated cod backbone, where IC_50_ values were 0.77 ± 0.03 and 0.53 ± 0.01 mg/mL, respectively [28]. The differences may be ascribed to the combination of a different substrate, lower pH, and temperature and higher enzyme concentration, which may have favored the production of iron chelating hydrolysates. Moreover, the fact that the controls of the enzyme-treated samples showed a similar or better iron chelating ability than the hydrolysates produced with enzymes suggests that the addition of enzymes did not favor the production of iron chelating peptides. Evidently, the autolysis that occurred during hydrolysis played a major role in producing hydrolysates with iron chelating properties. This indicates that it may be possible to produce hydrolysates with high iron chelating properties using heat treatment without addition of enzymes. Even though this action could lower the production cost of antioxidative hydrolysates by omitting the addition of enzymes also taking into account the lower quantities of supernatant obtained without enzyme addition (Table S1), more research should be conducted to investigate the probability of getting the same hydrolysate bioactivity in every hydrolysis batch. Earlier reported results on the antioxidant properties of enzymatic produced hydrolysates lack controls performed at the same hydrolysis conditions without enzyme addition to evaluate the effect of the addition of enzymes.

Several studies have evaluated the link between the amino acids and antioxidant activity of FPHs obtained from various sources, where aromatic amino acids (TYR, HIS, TRP, and PHE) and hydrophobic amino acids (VAL, LEU, and ALA) are mentioned to be critical for the antioxidative properties of the hydrolysates [38,39,40]. In the current study, the hydrolysates with the highest RSA (P_6hE and P_6hC) had a significantly higher content of HYP and GLU, whereas the hydrolysate with the highest iron chelating activity (N_6hC) had a significantly higher content of THR, LYS, TYR, and C-C. Thus, no differences in the hydrophobic amino acids and only significant differences in one of the aromatic amino acids for the total amino acids measured. However, not only the amino acid residues present but also the positioning within the peptide sequence plays an important role [2,13,41,42]. Therefore, it is assumed that the differences observed in the antioxidant activity for these samples are not only ascribed to the content of amino acids but must also be related to the sizes of the peptide in the hydrolysates and the sequences of the amino acids in the peptides, which has to be evaluated further.

The differences in peptide Mw distribution between controls and enzyme-treated samples could also explain the differences in antioxidative properties of the hydrolysates. Indeed, the hydrolysates with low molecular weight (10–1 and <1 kDa), as obtained by enzymatic treatments, could have improved RSA, with increased effect for the samples treated by Protamex, probably due to the higher presence of peptides in the band 10–1 kDa. On the other hand, the presence of hydrolysates with Mw between 200 and 10 kDa seemed to have a major effect on the iron chelating capacity, as described above, in which controls showed a slightly higher iron chelating capacity than Alcalase-treated samples and similar to Neutrase and Protamex-treated samples.

Liu et al. [43] obtained a similar molecular weight distribution for fish protein hydrolysates obtained by surimi by-products hydrolyzed by Alcalase and Protamex enzymes, finding both peptides with Mw higher than 150 kDa and lower than 10 kDa, with the latter being the major component for both the enzymes used. These results also agree with the study of Melgosa et al. [44], who extracted protein from cod backbones in different conditions. The authors described a similar molecular weight distribution with estimated Mw, which was divided into four bands: Mw > 1000 kDa, >300, 100–10, and <10 kDa. Also, in this case, the samples with the longest enzymatic treatment time presented the highest amounts of peptides with an estimated Mw around 1 kDa. However, in this case, the authors obtained these hydrolyzed peptides only through process conditions such as the temperature applied for protein extraction, highlighting the influence of processing conditions on the physicochemical characteristics of the extracted FPHs. In the current study, some samples were selected to compare differences observed for antioxidant activity with molecular weight distribution between different enzymes applied and without enzyme addition. Since the controls were different with respect to the molecular weight distribution, this suggests that some hydrolysis occurred without enzyme addition as highlighted by Melgosa et al. [44]. To improve the understanding of changes occurring with and without enzyme addition during the heating process, further analysis is needed for hydrolysates and raw material. For example, SDS page analysis, in addition to molecular weight distribution, could add valuable information.

### 3.3. Lack of Antimicrobial Activity

The fact that the hydrolysates were not purified could have influenced their antimicrobial activity. The size of the peptides and/or the hydrophobicity of the existing amino acids could have shown false negative results, due to the hydrolysates’ difficulty diffusing into the agar, and created the inhibition zones [45]. On the other hand, the produced hydrolysates may not have had antimicrobial activity against the tested strains, or they may not have any antimicrobial activity in general due to their size and/or amino acid sequence. A peptide with antimicrobial activity usually is medium-sized (12–15 amino acid residues [2,12]), which corresponds to the molecular fraction from 1 to 10 kDa. SEC-MALS results for hydrolysates produced without enzyme addition (controls) showed that only one treatment of the measured hydrolysates (N_6hC) had peptides in the molecular weight range of 10–1 kDa (Table 3). Even though the enzyme-produced hydrolysates also had peptides within this molecular weight range (10–1 kDa), antimicrobial activity was not detected in this study. This could indicate that not only the size but also the sequence of amino acids affects the properties of the produced hydrolysates [13]. However, the lack of activity could also be due to the selection of strains and assays applied as mentioned above, and this needs to be further evaluated and compared to the size of peptides in the produced hydrolysates for further conclusions. Moreover, other enzymes and process conditions could be applied in the search for hydrolysates with antimicrobial activity.

## 4. Materials and Methods

All enzymes used (Alcalase 2.4 AU-A/g, Neutrase: 0.8 AU-N/g and Protamex: 1.5 AU-N/g) were from Novonesis (Bagsværd, Denmark). Chemicals were of analytical grade, and solvents were of HPLC grade. Amino acid standards were purchased from Sigma-Aldrich (St. Louis, IL, USA). Butylated hydroxytoluene (BHT), ethylenediaminetetraacetic acid (EDTA), sodium dodecyl sulphate (SDS), ferrous chloride, sodium bicarbonate, DL-DiThioThreitol (DTT), ferrozine, and DPPH radical were obtained from Sigma-Aldrich (Steinheim, Germany). Sodium carbonate decahydrate obtained from Merck (Darmstadt, Germany).

### 4.1. Cod Fish

The Royal Greenland factory (Royal Greenland filleting factory, Maniitsoq, Greenland) received the cod fish alive and kept them alive until their slaughtering day. The fish were processed immediately after slaughtering, and the produced side-streams were collected and kept frozen (−20 °C) to preserve their quality for future applications. Cod backbones were shipped frozen (−20 °C) to Denmark, and, at the Technical University of Denmark (DTU), the cod backbones were kept at −40 °C until use.

### 4.2. Sample Preparation

Approximately 4.6 kg of cod backbones were weighed and defrosted overnight at refrigerator temperature. After defrosting, the backbones were chopped to produce a coarse mince using a high-speed blender for 2 min (Robot Coupe, Blixer 4, Brønnum, Herlev, Denmark). The coarse minced backbones were packed in plastic bags in small portions (13 pprox. 100–130 g mince) and stored at −80 °C until use. A portion of the coarse minced backbones was mixed with liquid nitrogen and blended into a homogeneous powder for protein determination prior to the hydrolysis. The protein content of the cod backbones was 12.6 ± 0.1%.

### 4.3. Hydrolysis

#### 4.3.1. Procedure for the Enzyme Hydrolysis

The coarse minced backbones were mixed with distilled water at 1:1 (*w*/*w*). For all treatments, controls (no enzyme added) and samples (enzyme addition) were included to evaluate the effect of the enzyme. The hydrolysis temperature was set to 60 °C for the Alcalase and Neutrase samples and to 50 °C for the Protamex samples. The pH (SI Analytics) of both the control and enzyme-treated samples was recorded and adjusted prior to the hydrolysis using 4 M NaOH and 2 M HCl (Table 1). When the samples reached their desired hydrolysis temperature, 1% of the enzyme was added based on the cod backbones’ protein content. For Alcalase, a protein conversion factor of 6.25 was applied, which corresponds to an enzyme concentration of 1.1% when calculated with a protein conversion factor of 5.58. This conversion factor has been shown to be more appropriate for raw fish materials [46]. The samples were hydrolyzed at different times (0.5–6 h) depending on the added enzyme (Table 1). To inactivate the added enzyme, the samples were heated to 90 °C and held for 15 min. The samples were cooled down to 22 °C and centrifuged (Sorvall RC 6+, ThermoFisher Scientific, Rockford, IL, USA) at 14000× *g* for 15 min. After centrifugation, the supernatant and precipitate were collected, weighed, and stored at −80 °C until further analysis. Only the supernatant was used for the current study and was freeze-dried and stored at −80 °C until further analysis. The hydrolysis process was performed in duplicates.

#### 4.3.2. Effect of Preheating or pH Adjustment Prior to Hydrolysis (Alcalase)

One set of samples for enzyme hydrolysis for 3 h using Alcalase was heated to 90 °C to inactivate the endogenous enzymes for 10 min after reaching the 90 °C prior to the hydrolysis. The samples were cooled to the hydrolysis temperature. Another set of samples for enzyme hydrolysis for 0.5 h using Alcalase was performed without pH adjustment prior to the hydrolysis. The rest of the hydrolysis process was similar to the one using Alcalase (Section 2.3.1).

### 4.4. Protein Determination

To determine the protein content, a Dumas (Rapid MAX N exceed cube N/protein analyzer, Elementar Analysensysteme GmbH, Langenselbold, Germany) was used. All samples were analyzed in duplicates (liquid and freeze-dried supernatant after hydrolysis), except from the fine minced cod backbones (raw material for the hydrolysis), which were in triplicate. To calculate the protein content of the samples, a protein factor of 5.58 was applied [46]. The protein content was displayed in % of sample weight.

### 4.5. Degree of Hydrolysis (DH) by the OPA Method

The o-phthaldialdehyde (OPA) reagent used was prepared daily (10 mL 0.15 M sodium carbonate decahydrate, 10 mL 0.6 M sodium bicarbonate, 10 mL 1% SDS, 88 mg DTT 99%, 80 mg OPA diluted in 2 mL 96% ethanol, and Milli-Q water). The supernatants were diluted to a protein concentration of 0.1% to 0.2% protein in Milli-Q water. The supernatants obtained from long hydrolysis time were diluted to 0.1% protein, whereas controls (no enzymes added) and supernatant obtained with short hydrolysis time were diluted to 0.2% protein. A total of 300 μL of vortexed diluted supernatant was added into a 96-well microtiter plate, where 3-step 2-fold dilutions were performed (directly in the plate). From each dilution, 20 μL of sample was transferred to a clean microtiter plate, and 200 μL of OPA reagent was added in each well. The microtiter plate was then put in the thermomixer (Eppendorf ThermoMixer C) for 15 s at 500 rpm, and the absorbance was immediately measured at 340 nm using a BioTek Eon microplate reader (Agilent Technologies, Santa Clara, CA, USA). DH was performed with analytical triplicates and calculated using a calibration curve with serine (mg/mL).

(1) $$mg\;serine/mL=\frac{\left(\left(Abs\;sample-Abs\;blank\right)-intercept\right)}{Slope}*DF$$

(2) $$DH\;(\%)\;=\frac{mg\;serine/mL*100\%}{P*10}$$

where Abs sample is absorbance of the sample, Abs blank is absorbance of blank, Slope and intercept are determined from the serine calibration curve, DF is the dilution factor, P is the protein content (%), and 10 converts % to mg/g.

### 4.6. Protein Extraction Yield (PEY)

The hydrolysis processes performed were evaluated by the protein extraction yield (PEY). The PEY was calculated based on protein content (g) in the supernatant divided with the initial protein content (g) of the sample (before hydrolysis) and reported in percentages (%).

(3) $$PEY\;\left[\%\right]\;\;=\;\;\frac{Protein\;content\;in\;the\;hydrolysates\;[g]}{Initial\;protein\;content\;in\;the\;sample\;[g]}\;\times 100$$

### 4.7. DPPH Radical Scavenging Capacity

The radical scavenging activity (RSA) of the produced hydrolysates was determined using the DPPH in vitro assay [47]. The samples were diluted in eight concentrations by 2-fold serial dilutions to determine IC_50_ (concentration for 50% inhibition). The concentration of the hydrolysates and dilutions was different due to different activities. At each concentration, 100 μL of each sample was transferred into a 96-well microtiter plate in triplicate, along with 100 μL of 0.1 mM DPPH solution. The microtiter plates were put in the thermomixer (Eppendorf ThermoMixer C) for 3 min at 500 rpm. The microtiter plates were incubated for 30 min at room temperature in dark conditions. Thereafter, the absorbance was measured at 517 nm using a BioTek Eon microplate reader (Agilent Technologies, Santa Clara, CA, USA). BHT was used as the positive control (0.2 mg/mL, inhibition 60–80%). Distilled water was used as blank, and methanol was added in the blind wells instead of DPPH. The inhibition percentages were calculated. The IC_50_ was calculated based on inhibition percentages below and above 50% inhibition by linear regression in a linear area 50% inhibition. All measurements were performed in triplicate.

### 4.8. Iron Chelation Capacity

The iron chelation activity of the produced hydrolysates was determined according to Farvin et al. [48] with modifications. The lyophilized samples were diluted to 10–20 mg/mL with distilled water. Due to increased turbidity, control samples of Alcalase were centrifuged (Biofuge pico) at 12,000× *g* for 5 min, and the supernatant was used for the next steps. The samples were further diluted to 8 different concentrations by 2-fold serial dilutions. Each concentration of each sample was transferred (100 μL) into a 96-well microtiter plate in triplicate, along with 110 μL of distilled water and 20 μL of 0.5 mM ferrous chloride. The microtiter plates were put in the thermomixer (Eppendorf ThermoMixer C, Hamborg, Germany) for 3 min at 500 rpm. Then, 20 μL of 2.5 mM ferrozine was added, and the plates were mixed for another 3 min. The microtiter plates were incubated for 10 min at room temperature and in dark conditions. Thereafter, the absorbance was measured at 562 nm using a BioTek Eon microplate reader (Agilent Technologies, Santa Clara, CA, USA). EDTA was used as the positive control (0.06 mM, chelating activity approx. 60%). Distilled water was used as blank, which was also added in the blind wells instead of ferrous chloride and ferrozine. The IC_50_ (concentration for 50% inhibition/chelation) was calculated based on iron chelation activity (%) below and above 50% by linear regression in the linear area around 50% activity. All measurements were performed in triplicate.

### 4.9. Total and Free Amino Acid Content (Selected Hydrolysates)

The amino acid composition (free and total) in samples was determined by liquid chromatography (LC)−MS. For total amino acid determination, the samples were first hydrolyzed and derivatized using the EZ:faast amino acid kit (Phenomenex, Torrance, CA, USA). Acid hydrolysis was applied under heat followed by neutralization and derivatization described by Fog [49]. For determination of free amino acids, the samples were only derivatized. Aliquots of the samples were injected into an Agilent HPLC 1100 instrument (Santa Clara, CA, USA) coupled to an Agilent ion trap mass spectrometer.

The total and free amino acids were identified by comparing retention time and mass spectra of an external standard mixture. Calibration curves were prepared and analyzed by HPLC–MS for quantification.

### 4.10. Molecular Weight by SEC-MALS (Selected Hydrolysates)

The molecular weight of the samples was determined using size-exclusion chromatography. A few hydrolysates were selected based on their antioxidant activity with the aim of evaluating the differences in size between samples produced with and without enzymes and their differences in activity. Mostly, it was the samples with the shortest hydrolysis time and the samples with the longest hydrolysis time from each enzyme treatment that were selected to evaluate the influence of the hydrolysis time on the molecular weight.

Lyophilized hydrolysates were prepared in phosphate buffer (pH 7.20) in a concentration of 2 mg/mL and filtered with 0.45 and 0.1 μm pore size filters before analysis on a HPLC. The HPLC (Agilent, Santa Clara, CA, USA) was equipped with WTC-015S5 column ((300 × 7.8mm, 150 Å maximum pore size); Wyatt Technology, Santa Barbara, CA, USA). The eluate was monitored in succession with a UV detector at 280 nm, a DAWN 8 light-scattering detector (Wyatt Technology, Santa Barbara, CA, USA), and an Optilab differential refractometer (Wyatt Technology). The flow rate was 0.8 mL/min, and the injection volume was 50 μL. The mobile phase was phosphate buffer, pH 7.20, containing 200 μL/L proClin (Sigma, St. Louis, MO, USA) to mitigate the microbial growth. The buffer was prefiltered with a sterile single-use vacuum filter (Thermo Fisher Scientific, Roskilde, Denmark) with a pore size of 0.1 μm. Specific-refractive-index increments (dn/dc) of sample solution was 0.185 mL/g. Data analysis and molecular weight calculations were performed using the ASTRA software (7.3.2 Version, Waters Corporation, Milford, MA, USA).

### 4.11. Antimicrobial Assays

#### 4.11.1. Culture Preparation

To determine the antimicrobial activity of the produced hydrolysates, the following strains were used: *Escherichia coli ATCC 25922* (American Type Culture College), *Salmonella typhimurium LT 2* (n.a.), *Salmonella paratyphi 13-SAO1718* (Bundesinstitut fur Risikowertung, Postfach), *Micrococcus luteus CCM 662* (Czech Collection of Microorganisms), and *Streptococcus suis tp2 SVS 321* (DTU Health Tech). The optimal density was adjusted to 1–2 × 10^8^ colony forming units (CFUs) in 0.9% saline suspension (NaCl).

#### 4.11.2. Minimum Inhibitory Concentration (MIC) Assay

For the MIC assay, the lyophilized hydrolysates were diluted to 10 and 100 mg/mL. The diluted hydrolysates were added in Mueller–Hinton II (MH II) broth in a microtiter plate and further diluted by 2-fold serial dilutions, followed by the addition of the inoculum. The positive control was MH II broth with the inoculum, and the sterile control was only MH II broth. The microtiter plates were incubated at 37 °C for 18–24 h depending on the strain used. The first well (from low to high hydrolysate concentration) with non-bacterial growth was determined as the MIC via visual observation.

#### 4.11.3. Disk Diffusion Assay

For the disk diffusion assay, the lyophilized hydrolysates were diluted to 200 mg/mL. The diluted sample was suspended on a sterile cotton disk (OXOID), which was left to dry for 30 min. MH II agar plates were streaked with E. coli ATCC 25922, S. typhimurium LT 2, and S. paratyphi 13-SAO1718, and brain heart infusion (BHI) agar plates were streaked with M. luteus CCM 662 and S. suis tp2 SVS 321. The cotton disks were placed on the plates. The MH II agar plates were incubated at 37 °C for 18 h (±1 h), while the BHI agar plates were incubated at 37 °C for 24 h. The diameter of the clear/inhibition zones around the disks was measured to determine the antimicrobial activity.

### 4.12. Statistical Analysis

Results for the different analysis are reported as average and standard deviation. The program Statgraphic (Version 18.1.08, Statpoint Technologies, Inc., Warrenton, VA, USA) was applied for statistical analysis. Mean value, standard deviation, and number of replicates were used for the analysis of variance (ANOVA). Multiple sample comparison was performed to identify significant differences between sample treatments followed by Tukey’s post-test. The threshold of significance was set at 5%.

## 5. Conclusions

Cod backbone, a side-stream from cod filleting, can be utilized for the production of hydrolysates with biocative properties since the application of Alcalase, Neutrase, or Protamex resulted in hydrolysates with radical scavenging and iron chelation activities. Interestingly, the iron chelation activity was not higher for enzymatic produced hydrolysates compared to only a heat treatment for the extraction of the proteins. Thus, the production costs of these hydrolysates can be reduced since enzymes can be left out. However, the protein extraction yield was lower without enzyme addition compared to Alcalase and Protamex (2–6 h)-treated hydrolysates.

Based on the molecular weight of the selected hydrolysates, both enzyme-treated and controls, it seems like the smaller hydrolysates <10 kDa are less important for iron chelation activity since the size of the peptide in the controls was 200–1 kDa, whereas smaller-sized peptides in the hydrolysates (10–≤ 1) resulted in higher radical scavenging activity. There was no relation between specific amino acid composition and antioxidant activity, suggesting that the size and sequence influence the activity. The evaluated enzyme treatments did not result in any hydrolysates with antimicrobial activity despite the use of different enzymes and hydrolysis times to produce hydrolysates with a broad range of peptides. In the search of hydrolysates with antimicrobial activity, other enzymes or hydrolysis conditions can be evaluated or other bacterial strains in future research.

## Figures and Tables

**Table 1:** Experimental design for the hydrolysis process using different enzymes: Alcalase (A), Neutrase (N), and Protamex (P).

Enzyme Applied ^1^	Hydrolysis Condition			
	Time (h) ^2^	pH (Adjusted)	Temperature (°C)	Preheating
Alcalase (A)	0.5, 1, 2, 3	8.0	60	-
Alcalase (A_pHn)	0.5	-	60	-
Alcalase (A_P)	3	8.0	60	15 min, 90 °C
Neutrase (N)	0.5, 1, 2, 3, 6	8.0	60	-
Protamex (P)	0.5, 1, 2, 3, 6	6.5	50	-

^1^ Treatments with enzymes will have an E in the end of the code, whereas treatment without enzyme addition (control) will have a C in the of the code, i.e., A_1hE (with Alcalase) and A_1hC (without Alcalase). ^2^ For samples processed for 0.5 h, ½ h is used for code names.

**Table 2:** Total and free amino acids (mg/g) measured in three selected hydrolysates, Protamex 6h (P_6hE), control from Protamex treatment (P_6hC), and control from Neutrase treatment 6 h (N_6hC). Different letters in superscript (a–c) for the individual total and free amino acids indicate significant differences between hydrolysates (n = 4).

Free Amino Acids [mg/g] ^1^				Total Amino Acids [mg/g] ^1^			
	P_6hE	P_6hC	N_6hC		P_6hE	P_6hC	N_6hC
**ARG**	16.5 ± 3.2 ^c^	6.3 ± 2.2 ^b^	1.4 ± 0.1 ^a^	**ARG**	56.6 ± 3.9 ^b^	49.0 ± 4.4 ^a^	51.6 ± 2.4 ^ab^
**SER**	3.6 ± 0.3 ^a^	3.9 ± 1.0 ^a^	3.1 ± 0.4 ^a^	**SER**	37.7 ± 2.5 ^b^	32.3 ± 2.3 ^a^	33.1 ± 2.9 ^ab^
**HYP**	6.2 ± 0.9 ^c^	2.5 ± 0.5 ^b^	1.0 ± 0.0 ^a^	**HYP**	33.2 ± 4.6 ^b^	26.8 ± 3.2 ^b^	17.6 ± 2.6 ^a^
**GLY**	4.2 ± 0.5 ^c^	3.0 ± 0.6 ^b^	1.4 ± 0.2 ^a^	**GLY**	108 ± 17 ^b^	101 ± 24 ^b^	64.7 ± 9.0 ^a^
**THR**	3.8 ± 0.6 ^c^	1.5 ± 0.3 ^b^	0.6 ± 0.0 ^a^	**THR**	19.6 ± 1.0 ^a^	18.0 ± 2.2 ^a^	23.6 ± 1.8 ^b^
**ALA**	16.4 ± 1.6 ^c^	9.7 ± 3.0 ^b^	4.1 ± 0.6 ^a^	**ALA**	68.9 ± 5.0 ^b^	61.5 ± 11 ^ab^	48.0 ± 4.6 ^a^
**PRO**	0.5 ± 0.1 ^ab^	0.7 ± 0.2 ^b^	0.3 ± 0.1 ^a^	**PRO**	57.9 ± 5.3 ^a^	55.3 ± 11 ^a^	41.9 ± 6.9 ^a^
**MET**	6.9 ± 0.7 ^c^	1.5 ± 0.3 ^b^	0.4 ± 0.0 ^a^	**MET**	16.6 ± 2.9 ^a^	15.2 ± 3.2 ^a^	17.1 ± 1.7 ^a^
**ASP**	3.5 ± 0.9 ^c^	1.8 ± 1.0 ^b^	0.3 ± 0.0 ^a^	**ASP**	49.6 ± 1.4 ^a^	49.6 ± 8.4 ^a^	58.8 ± 7.3 ^a^
**VAL**	8.9 ± 1.4 ^b^	1.6 ± 0.6 ^a^	0.4 ± 0.2 ^a^	**VAL**	24.2 ± 1.2 ^a^	24.8 ± 5.6 ^a^	29.7 ± 3.5 ^a^
**HIS**	4.4 ± 0.7 ^b^	0.5 ± 0.1 ^a^	0.1 ± 0.0 ^a^	**HIS**	11.8 ± 1.5 ^a^	13.2 ± 3.3 ^a^	15.1 ± 2.3 ^a^
**LYS**	4.6 ± 1.1 ^b^	0.6 ± 0.3 ^a^	0.3 ± 0.1 ^a^	**LYS**	47.0 ± 2.7 ^a^	43.3 ± 5.8 ^a^	63.5 ± 9.7 ^b^
**GLU**	4.3 ± 0.6 ^b^	3.3 ± 2.0 ^b^	0.9 ± 0.0 ^a^	**GLU**	82.6 ± 4.5 ^a^	70.3 ± 11 ^a^	84.0 ± 10 ^a^
**TRP**	1.2 ± 0.2 ^b^	0.2 ± 0.0 ^a^	0.1 ± 0.0 ^a^	**TRP**	0.2 ± 0.1 ^a^	0.2 ± 0.3 ^a^	0.2 ± 0.2 ^a^
**LEU**	6.9 ± 1.3 ^c^	3.0 ± 0.5 ^b^	0.5 ± 0.2 ^a^	**LEU**	36.5 ± 5.4 ^a^	31.3 ± 6.2 ^a^	37.8 ± 2.8 ^a^
**PHE**	8.0 ± 0.7 ^b^	1.2 ± 0.1 ^a^	0.4 ± 0.0 ^a^	**PHE**	16.7 ± 3.4 ^a^	18.3 ± 4.1 ^a^	19.3 ± 2.0 ^a^
**ILE**	8.0 ± 1.0 ^c^	3.5 ± 0.6 ^b^	0.7 ± 0.3 ^a^	**ILE**	19.9 ± 2.8 ^a^	19.4 ± 3.9 ^a^	25.0 ± 4.0 ^a^
**C-C**	0.1 ± 0.0			**C-C**	1.8 ± 0.1 ^a^	2.4 ± 0.3 ^b^	4.4 ± 0.3 ^c^
**TYR**	4.2 ± 0.5 ^c^	1.1 ± 0.2 ^b^	0.5 ± 0.0 ^a^	**TYR**	6.1 ± 0.6 ^a^	9.2 ± 1.3 ^b^	14.0 ± 1.9 ^c^
**SUM**	112 ± 15 ^c^	45.8 ± 13 ^b^	16.4 ± 1.2 ^a^	**SUM**	695 ± 57 ^a^	641 ± 103 ^a^	649 ± 70 ^a^

^1^ Abbreviation of the amino acids: ARG Arginine; SER Serine; HYP 4-hydroxyproline; GLY Glycine; THR Threonine; ALA Alanine; PRO Proline; MET Methionine; ASP Aspartic acid; VAL Valine; HIS Histidine; LYS Lysine; GLU Glutamic acid; TRP Tryptophan; LEU Leucine; PHE Phenylalanine; ILE Isoleucine; C-C Cystine; TYR Tyrosine.

**Table 3:** Relative proportion (%) of each molecular weight (Mw) band of the hydrolysates obtained by SEC-MALS Chromatography. For sample treatment, refer to Table 1.

Sample ^1^		Mw (kDa)				
		>200	200–100	100–10	10–1	≤1
**No Enzymes, Control (C)**	**A_½ hC (N_½ hC)**		49.1	50.9		
	**A_pHn_½ hC**		45.0	55.0		
	**P_½ hC**		26.5	73.5		
	**A_P_3hC**		41.8	58.2		
	**N_6hC**		47.6	28.0	24.4	
**Enzyme Added (E)**	**N_6hE**	1.8		29.1	53.2	15.9
	**P_6hE**				15.4	84.6
	**A_P_3hE**	0.3		1.5	3.2	95.0
	**A_3hE**	0.3		0.6	2.1	97.0

^1^ Abbreviation in the sample treatments. A: Alcalase; N: Neutrase; P: Protamex; pHn: no pH adjustment prior to hydrolysis; _P: preheating prior to the hydrolysis to inactivate endogenous enzymes; Xh: time of treatment in hours; C: control, no enzymes added; E: enzyme added. Examples of codes: N_6hC is hydrolyzed for 6 h without enzyme under Neutrase condition, and N_6hE is hydrolyzed with Neutrase for 6 h.

## Data Availability

The data presented in this study are available on request from the corresponding author.

## Supplementary Materials

The following are available online at https://www.mdpi.com/article/10.3390/md23030125/s1: Table S1. Amount of supernatant (freeze-dried, g) and protein (g) obtained applying the different hydrolysis treatments (enzyme, temperature, time, and pH). For conditions of treatments, refer to Table 1. Figure S1. Chromatogram obtained from SEC-MALS. Each sample is a different colored line: (A1) A_½ hC /N_½ hC, (A2) A_pHn_½ hC, (A3) P_½ hC, (A4) A_P_3hC, (A5) N_6hC, (A6) N_6hE, (A7) P_6hE, (A8) A_P_3hE, and (A9) A_3hE. For sample abbreviations, refer to Table 1.
