# Supplementary material for: Production of Protein Hydrolysates from Cod Backbone Using Selected Enzymes: Evaluation of Antioxidative and Antimicrobial Activities of Hydrolysates
Source: Mar Drugs. 2025 Mar 13;23(3):125. doi: 10.3390/md23030125 (PMC11944100; doi:10.3390/md23030125)
Supplement: Supplementary file 1 [file marinedrugs-23-00125-s001.zip › marinedrugs-3503836-supplementary.pdf]

Table S1. Amount of supernatant (freeze dried, g) and protein (g) obtained applying the different hydrolysis treatments (enzyme, temperature, time and pH). For conditions of the treatments refer to Table 1.

	**Sample**	**Supernatant, Freeze dried [g]**	**Protein [g]**
ALCALASE	½hC	2.83 ± 0.24	2.11 ± 0.20
	½hE	4.38 ± 0.02	3.32 ± 0.08
	pHn_½hC	2.53 ± 0.08	1.95 ± 0.08
	pHn_½hE	4.09 ± 0.11	3.15 ± 0.10
	1hC	3.43 ± 0.26	2.60 ± 0.29
	1hE	5.01 ± 0.13	3.86 ± 0.13
	2hC	2.97 ± 0.29	2.22 ± 0.28
	2hE	5.05 ± 0.17	3.84 ± 0.13
	3hC	3.20 ± 0.02	2.39 ± 0.03
	3hE	5.16 ± 0.41	4.00 ± 0.29
	P_3hC	3.16 ± 0.19	2.42 ± 0.19
	P_3hE	5.17 ± 0.27	4.00 ± 0.25
NEUTRASE	½hC	2.83 ± 0.24	2.11 ± 0.20
	½hE	2.15 ± 0.39	1.71 ± 0.31
	1hC	2.53 ± 0.08	1.95 ± 0.08
	1hE	2.45 ± 0.19	2.07 ± 0.22
	2hC	2.97 ± 0.29	2.22 ± 0.28
	2hE	2.49 ± 0.15	2.00 ± 0.09
	3hC	3.20 ± 0.02	2.39 ±0.03
	3hE	2.36 ± 0.10	1.92 ±0.12
	6hC	3.31 ± 0.21	2.79 ± 0.22
	6hE	2.66 ± 0.27	2.18 ± 0.21
PROTAMEX	½hC	2.99 ± 0.24	2.56 ± 0.29
	½hE	3.25 ± 0.02	2.71 ± 0.06
	1hC	2.67 ± 0.09	2.23 ± 0.11
	1hE	3.52 ± 0.05	2.92 ± 0.05
	2hC	2.78 ± 0.01	2.31 ± 0.01
	2hE	4.19 ± 0.02	3.51 ± 0.02
	3hC	2.78 ± 0.06	2.34 ± 0.07
	3hE	3.93 ± 0.41	3.27 ± 0.34
	6hC	3.13 ± 0.17	2.67 ± 0.17
	6hE	4.34 ± 0.29	3.56 ± 0.28

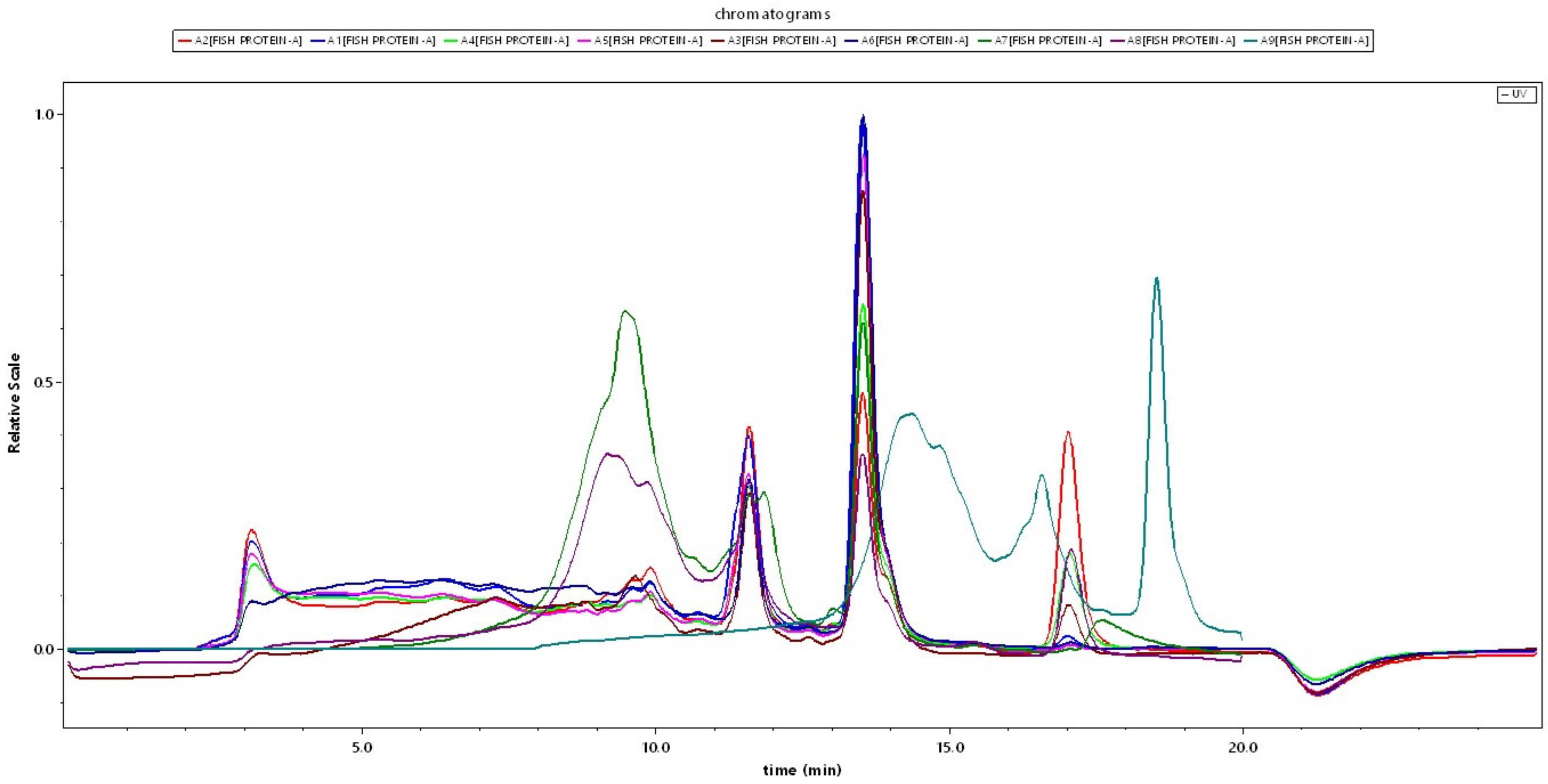

Figure S1. Chromatogram obtained from SEC-MALS. Each sample is a different colored line: A1) A_½hC / N_½hC, A2) A_pHn_½hC, A3) P_½hC, A4) A_P_3hC, A5) N_6hC, A6) N_6hE, A7) P_6hE, A8) A_P_3hE and A9) A_3hE. For sample abbreviations refer to Table 1.
